# Molecular Targeting of Carbonic Anhydrase IX in Mice with Hypoxic HT29 Colorectal Tumor Xenografts

**DOI:** 10.1371/journal.pone.0010857

**Published:** 2010-05-27

**Authors:** Sean Carlin, Nahida Khan, Thomas Ku, Valerie A. Longo, Steve M. Larson, Peter M. Smith-Jones

**Affiliations:** 1 Department of Radiology, Memorial Sloan Kettering Cancer Service, New York, New York, United States of America; 2 Department of Medical Physics, Memorial Sloan Kettering Cancer Service, New York, New York, United States of America; Genentech, United States of America

## Abstract

**Background:**

Carbonic anhydrase IX (CAIX) is a membrane spanning protein involved in the enzymatic regulation of tumor acid-base balance. CAIX has been shown to be elevated in a number of hypoxic tumor types. The purpose of this study was to determine the efficiency of intact and IgG fragments of cG250 to target CAIX *in vivo* in a hypoxic tumor model.

**Methodology/Principal Findings:**

Conventional biodistribution studies were performed with ^111^In-DO3A-cG250,^ 111^In-DO3A-F(ab')_2_-cG250 and^ 111^In-DO3A-Fab-cG250. Additional *ex vivo* analysis of the tumor was performed with markers for tumor hypoxia, blood perfusion and endogenous CAIX expression. All four data sets were digitally correlated to determine the optimal agent for determining hypoxia in a HT29 colon cancer xenograft. The HT29 human colorectal tumor xenografts show strong CAIX expression in hypoxic areas of poor blood perfusion. The intact IgG had an initial high focal uptake at the periphery of these hypoxic regions and penetration into the areas of highest CAIX expression over the 7-day study period. The lower molecular weight antibody fragments had a faster uptake into areas of high CAIX expression, but had a much lower absolute uptake at the optimal imaging times.

**Conclusions/Significance:**

For the clinical detection of hypoxia induced CAIX using cG250 antibody based agents, imaging with the intact IgG at 7 days post injection would allow for the most sensitive and accurate detection of CAIX.

## Introduction

Carbonic Anhydrase 9 (CAIX, G250/MN) is a membrane-spanning protein involved in the enzymic regulation of tumor acid-base balance (reviewed by [1]). Immunohistochemical determination of CAIX has revealed elevated expression in an increasing number of diverse tumor types including those of kidney, breast, bladder, head and neck, cervix, soft tissue sarcoma and in non-small cell lung carcinoma [2], [3], [4], [5], [6], [7]. Malignancies of the gastrointestinal tract have been the most widely studied, with high CAIX expression observed in esophageal, hepatobiliary, pancreatic, gastral and colorectal tumors [8], [9], [10]. The elevated expression of CAIX is restricted to malignant tissue, with normal tissue expression restricted to epithelia of the stomach, small intestine and gall bladder [1], [11].

Reduced pO_2_ (hypoxia) is a phenomenon of solid tumors resulting from an insufficient vascular network, and has been associated with tumor propagation, malignant progression and resistance to chemo- and radiotherapy in many tumor types [12]. Hypoxia results in an increase in the level of expression of Hypoxia-Inducible Factor 1α (HIF-1α), which, as part of the dimeric transcription factor HIF1, regulates the expression of a large number of genes involved in cell proliferation apoptosis, glucose metabolism, pH regulation and angiogenesis [13]. The expression of CAIX is regulated by HIF1 and is strongly-inducible under hypoxic conditions [14]. This has led to suggestions that CAIX expression may serve as an endogenous marker of tumor hypoxia [1]. Whilst pre-clinical and early clinical studies have shown strong correlations between CAIX expression and tumor hypoxia (assessed by both Eppendorf pO2 probe measurements and the exogenous hypoxia tracer pimonidazole) [6], [15], [16], the general usefulness of CAIX as an endogenous marker of tumor hypoxia remains to be fully established. The elevated expression of CAIX, however, has been independently associated with poor prognosis in a growing number of tumor types, including those of breast, lung, cervix, head and neck, rectal and brain [3], [5], [6], [10], [17], making it an attractive target for diagnostic non-invasive imaging and also as a potential biomarker of treatment response.

CAIX is also constitutively expressed at high levels in clear cell renal carcinoma (ccRCC), due an inactivating mutation in the Von Hippel Landau E3 ligase protein (VHL). VHL mutation results in persistently elevated HIF1a expression, and subsequent upregulation of HIF-regulated genes, including CAIX [18]. Clinical studies have previously established high CAIX expression as a diagnostic and prognostic indicator in ccRCC (reviewed by [19]).

The chimeric anti-CAIX antibody cG250, radiolabeled with ^124^I, has completed phase I testing and is currently under multi-center clinical assessment as a PET diagnostic agent for ccRCC [20]. The purpose of this study was to generate low molecular weight Fab and F(ab')_2_ fragments of the CAIX-targeted chimeric antibody cG250, to compare their CAIX binding affinity to that of intact cG250 and to evaluate their potential as imaging agents in a xenograft tumor model with heterogeneous CAIX expression, as a prelude to using one of these agents for clinical imaging CAIX in hypoxic tumors.

## Results

An average of 5.2±0.2, 4.1±0.2 and 1.9±0.1 molecules of 1,4,7,10-tetraazacyclododecane-N, N',N'',N'''-tetraacetic acid (DOTA) were conjugated to the IgG, F(ab')_2_ and Fab forms of cG250. This represents a w/w DOTA loading of 1.3, 1.3 and 1.2%. Specific activities of 370 MBq/mg were easily achieved with labeling efficiencies of >90% and chemical purity of >99.9%. The immunoreactivity of these radiolabeled antibodies was preserved at >90% (Figure S1). The binding affinity for CAIX expressed by SKRC-38 cells was 2.5 and 1.8 nM for the IgG and F(ab')_2_ forms and 14 nM for the Fab fragment. The saturation binding data for all three forms of cG250 gave around 1.2×10^6^ sites/cells (Figures S1A, S1B and S1C).

**Figure 1:**
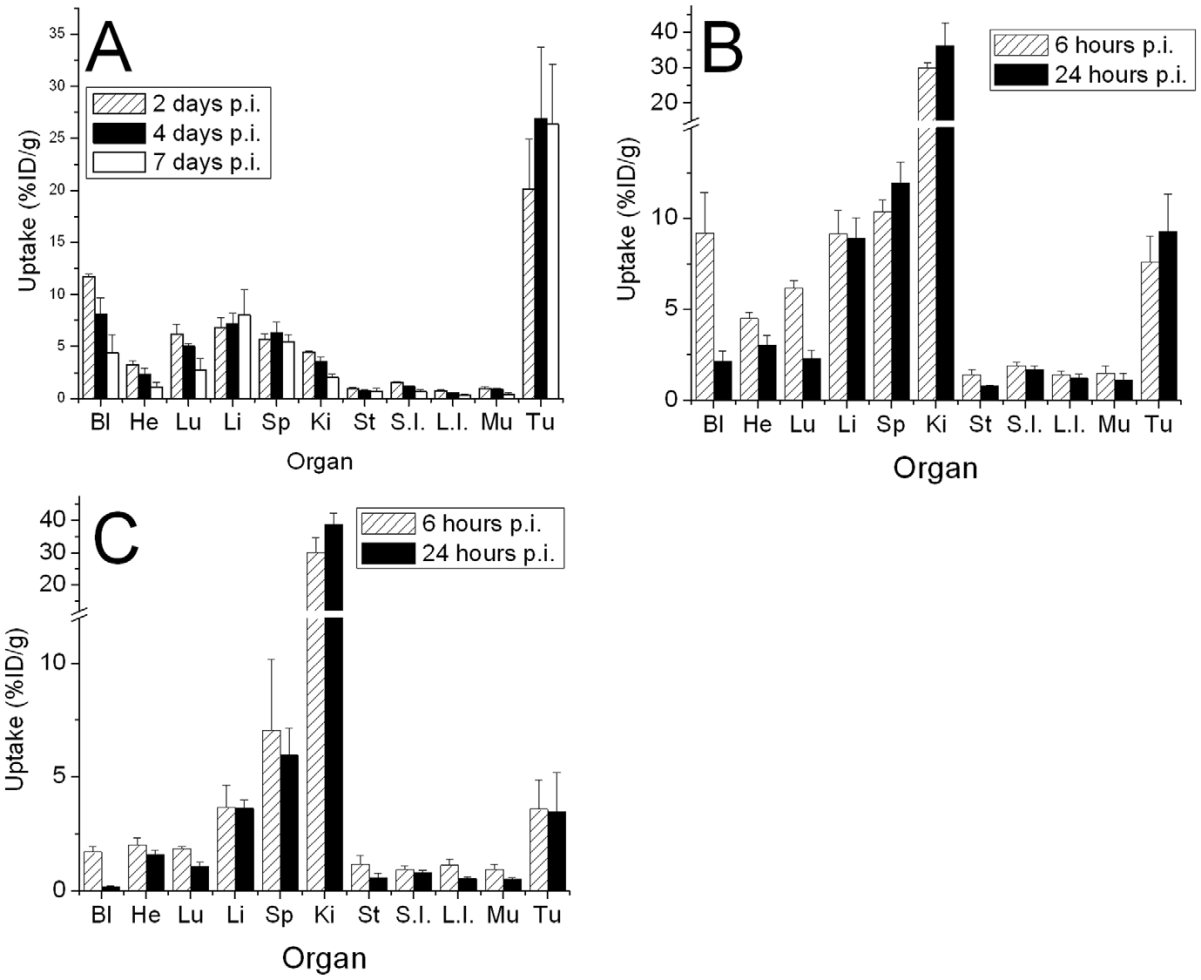
Biodistribution of ^111^In labeled intact and fragmented cG250 in athymic mice with hypoxic HT29 colorectal tumors. A: ^111^In-DOTA-cG250. B: ^111^In-DOTA-F(ab')_2_-cG250. C: ^111^In-DOTA-Fab-cG250. Legend Bl: blood, He: heart, Lu: lungs, Sp: spleen, Ki: kidneys, St: stomach, S.I.: small intestine, L.I.: large intestine, Mu: muscle and Tu: tumor.

The biodistribution of ^111^In-DOTA-cG250 in nude mice with HT29 tumors (Figure 1A) showed an initial high uptake of radioactivity (20.1±4.8%ID/g) at 2 days p.i.. However, the biodistribution of two similarly-labeled irrelevant antibodies (J591 and 3S193) also showed relatively high uptakes of 7.4±2.8 and 8.1±2.7%ID/g (data not shown). The biodistribution in all other organs was similar for all three radiolabeled IgGs. The uptake of the cG250 IgG increased marginally to 26.9±6.8 and 26.4±5.7%ID/g at days 4 and 7 p.i. (Figure 1A). By comparison the uptake by the irrelevant antibodies decreased to 1.7±1.1 and 3.8±0.8 for ^111^In-J591 and ^111^In-3S193. The activity slowly cleared from all organs except the liver and spleen and this clearance mimicked the overall blood clearance. Similarly the tumor/non tumor ratios increased over the 7 day period for all organs except liver and spleen. By 7 days post injection, the tumor/muscle ratio was 69 (Figure S2A). The tumor/blood ratio was moderate at 6.6.

The radiolabeled F(ab')_2_ fragment of cG250 showed lower absolute tumor uptakes of 7.6±1.4 and 9.3±2.1%ID/g at 6 and 24 hours post injection (Figure 1B). The tumor/muscle ratio was only 8.9 at 24 hours p.i. compared to 69 for the IgG at 7 days p.i.. The tumor/blood ratios were lower than the IgG and were 0.9 and 4.6 at 6 and 24 hours p.i. (Figure S2B).

The Fab fragment had the lowest tumor uptake of 3.6±1.3 and 3.5±1.7%ID/g at 6 and 24 hours post injection (Figure 1C). This low tumor uptake is due to its much faster clearance from circulation (0.20±0.02%ID/g blood at 24 h p.i.). At 6 hours p.i. tumor/muscle ratios were 4.8, but the tumor/blood ratios were still poor at 2.8 (Figure S2C) and similar to the IgG at 4 days p.i. At 24 hours p.i. tumor/muscle ratios had increased to 6.7., but the tumor/blood ratios were 16.6 (Figure S2C) and higher than either the IgG at 7 days or the F(ab)_2_ at 24 hours p.i..

**Figure 2:**
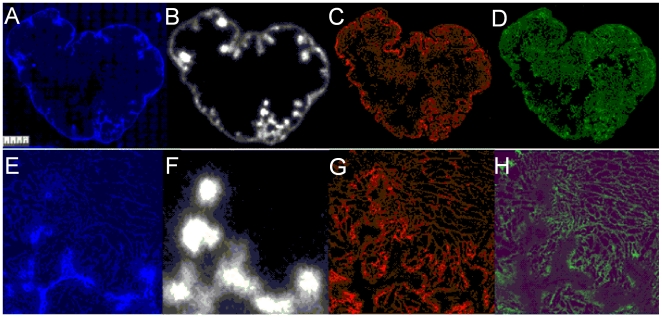
Images of the same HT29 tumor section showing blood perfusion, *in vivo* targeting of CAIX by ^111^In-DOTA-cG250, endogenous CAIX expression and hypoxia at 2 days p.i.. A: blood perfusion (*in vivo* targeted with Hoechst, blue), B: *in vivo* targeted CAIX (^111^In-DOTA-cG250, white), C: endogenous CAIX (*ex vivo* cG250 immunofluorescence, red) and D: hypoxia (*in vivo* targeted with pimonidazole, green) at 2 days pi. Bottom panel : Enlarged 3×3 mm areas of tumor sections showing E: blood perfusion (*in vivo* targeted with Hoechst, blue), F: *in vivo* targeted CAIX (^111^In-DOTA-cG250, white), G: endogenous CAIX (*ex vivo* cG250 immunofluorescence, red) and H: hypoxia (*in vivo* targeted with pimonidazole, green).

**Figure 3:**
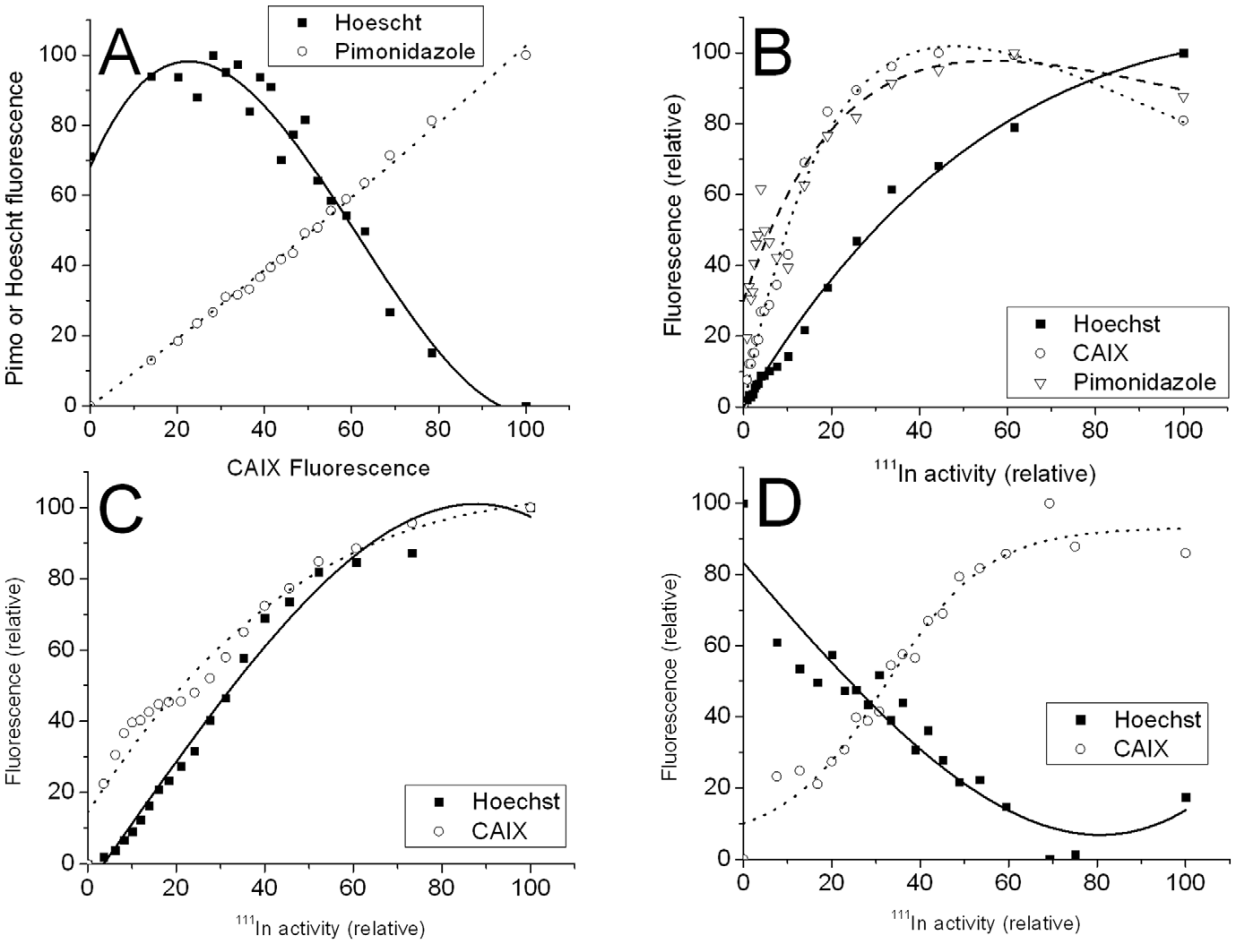
Binned pixel by pixel analysis of ^111^In-DOTA-cG250 distribution, tumor hypoxia, endogenous CAIX expression and blood perfusion in single HT29 tumor sections. A: Typical correlation hypoxia with endogenous CAIX expression and anticorrelation with tumor perfusion. B: Correlation at 2 days p.i. of ^111^In-DOTA-cG250. C: Correlation at 4 days p.i. of ^111^In-DOTA-cG250. D: Correlation at 7 days p.i. of ^111^In-DOTA-cG250.

Macroscopic examination of the tumor sections at 2 days post injection of ^111^In-cG250 (Figure 2) shows areas of high perfusion around the rim of the tumors as well as discrete areas within the core (blue). The pimonidazole and endogenous CAIX staining (green and red) are generally in good agreement with some minor variations which presumably arise from short-lived fluctuations in tumor perfusion and slow rates of CAIX degradation. The graphical correlation of endogenous CAIX and pimonidazole staining in Figure 2 was linear with a slope of 1 (c.f. Figure 3A) illustrating that the endogenous CAIX expression is restricted to hypoxic regions of the tumor. Similarly there was a negative correlation between the tumor perfusion and CAIX expression (Figure 3A).

These images also show an initial heterogenous and focal uptake of radioactivity at 2 days p.i. (Figure 2) which progressively becomes more diffuse over the 7 day period (see Figures S3 and S4). The enlarged microscopic views (Figure 2) show the anti-correlation between the Hoechst perfusion marker (blue) and the endogenous CAIX (red). The pixel by pixel analysis of this tumor section (Figure 3B) graphically illustrates the correlation of ^111^In activity and perfusion (Hoechst) in the tumor with a slope approaching 1 at 2 days post injection. The correlation between ^111^In and CAIX (and pimonidazole) is quite flat at high ^111^In levels indicating that there is some targeting of CAIX fairly close to the blood vessels, but this uptake is not directly proportional to the endogenous CAIX expression. At 4 and 7 days post injection the sections show a more diffuse pattern of ^111^In activity (Figure S3 and S4). The digital analysis (Figure 3C and 3D) clearly show the progressive clearance of ^111^In from well perfused areas of the tumor and the slope of the curve approached -1 at 7 days postinjection. The correlation of ^111^In with endogenous CAIX improves over time and the wider spacing between the binned ^111^In data points is indicative of a more diffuse distribution of radioactivity within the tumor section. These findings confirm the generally accepted view that IgGs and large molecules can have early high uptakes at the tumor due to the tumor vasculature being permeable to such large molecules. However these data show that by 7 days post injection the antibody has diffused away from the tissue adjacent to the vasculature and moved to the poorly perfused hypoxic regions rich in CAIX.

**Figure 4:**
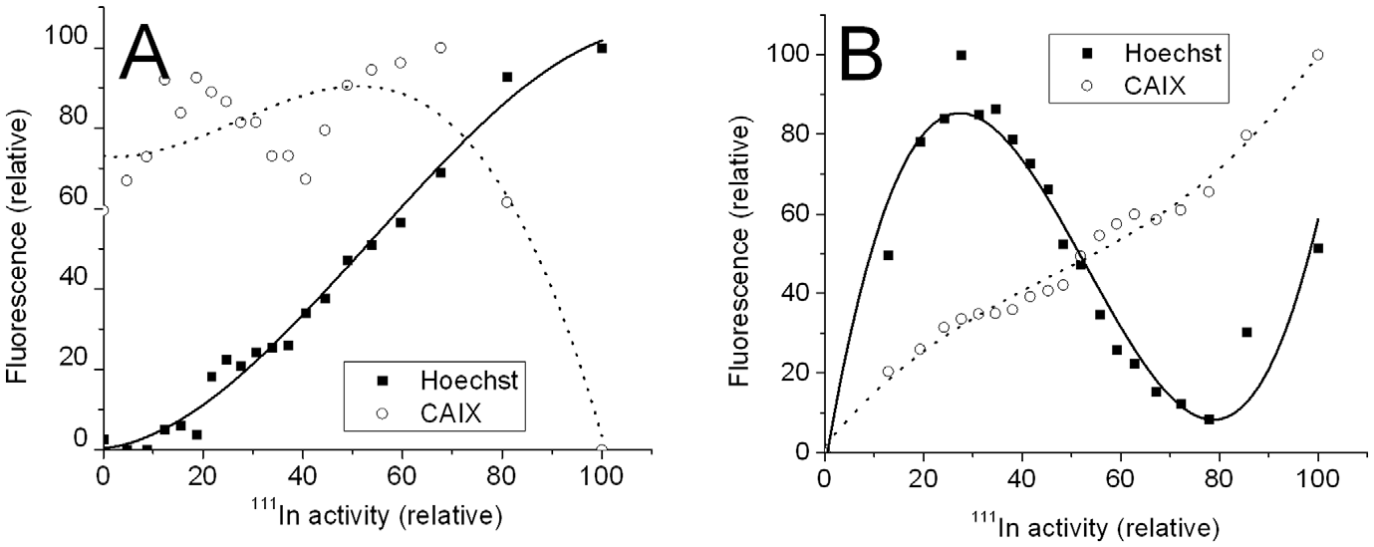
Binned pixel by pixel analysis of ^111^In-DOTA-F(ab')_2_-cG250 distribution, endogenous CAIX expression and blood perfusion in single HT29 tumor sections. A: Correlation at 6 hours p.i. of ^111^In-DOTA-F(ab')_2_-cG250. B: Correlation at 24 hours p.i. of ^111^In-DOTA-F(ab')_2_-cG250.

The two control antibodies at 7 days p.i. (Figures S5 and S6) showed some activity in well perfused tumor regions, little activity in hypoxic CAIX regions, but also significant amounts of radioactivity in tumor regions which were furthest away from the vasculature. The pixel by pixel analysis of the data (Figure S7) showed an inverse relationship between ^111^In uptake and CAIX immunofluorescence which was the inverse of that seen with the CAIX-specific cG250 (Figure 4D). The correlation between ^111^In activity and tumor perfusion was comparable to the early cG250 data at 2 and 4 days p.i. (Figure 4B and 4C). because most of the activity was still in the well perfused tumor regions.

The analysis of the fluorescent images for the tumor sections removed from animal injected with ^111^In-F(ab')_2_-cG250 at 6 and 24 hours post injection (Figures S8 and S9) is shown in Figures 4A and 4B. For the 6 hour p.i. image, the distribution of ^111^In and the correlation with perfusion and endogenous CAIX is similar to that seen with the intact IgG at 2 day p.i.. At 24 hours p.i., the ^111^In is almost ideally coregistered with the endogenous CAIX and there is almost an inverse correlation with Hoechst staining, except for the highest ^111^In data points. These data show that the distributions of ^111^In-F(ab')_2_-cG250 at 24 hours p.i. is comparable to the intact IgG at 7 days post injection with excellent targeting of CAIX, but some residual activity in well perfused and non-hypoxic areas. These data reflect the fact that the F(ab')_2_ fragmented antibody clears more rapidly from the blood and can more rapidly diffuse into hypoxic area rich in CAIX than the intact IgG, but at the optimum time the absolute tumor uptake of the F(ab')_2_ fragment is around 3 times lower and non specific uptake by the liver, spleen and kidneys is considerably higher.

**Figure 5:**
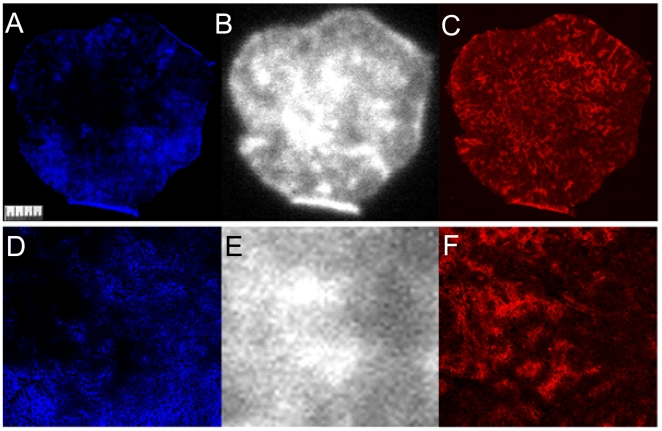
HT29 tumor sections showing blood perfusion, *in vivo* targeting of CAIX by ^111^In-DOTA-Fab-cG250 and endogenous CAIX expression at 24 hours pi. A: blood perfusion (*in vivo* targeted with Hoechst, blue), B: *in vivo* targeted CAIX (^111^In-DOTA-Fab-cG250, white) and C: endogenous CAIX (*ex vivo* cG250 immunofluorescence, red). Enlarged 3×3 mm areas of tumor sections showing; D: blood perfusion (*in vivo* targeted with Hoechst, blue), E: *in vivo* targeted CAIX (^111^In-DOTA-Fab-cG250, white) and F: endogenous CAIX (*ex vivo* cG250 immunofluorescence, red).

**Figure 6:**
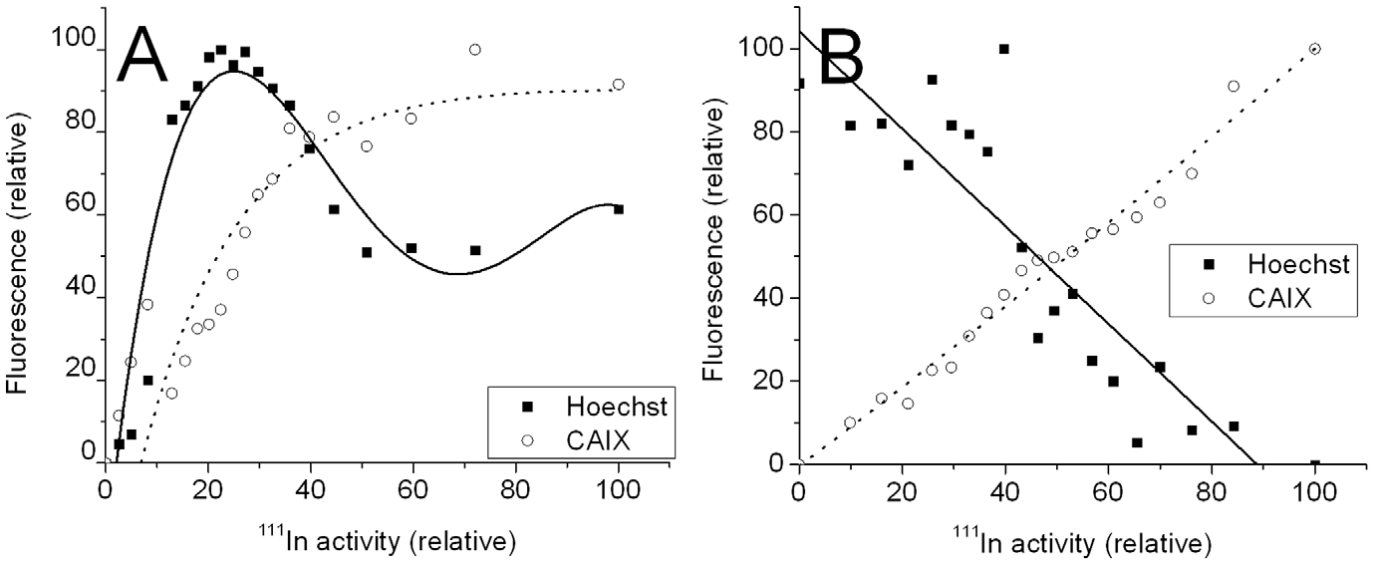
Binned pixel by pixel analysis of single HT29 tumor sections showing the correlation of *in vivo* targeting of CAIX by ^111^In-DOTA-Fab-cG250 with endogenous CAIX expression and blood perfusion. A; Correlation at 6 hours p.i. of ^111^In-DOTA-Fab-cG250. B: Correlation at 24 hours p.i. of ^111^In-DOTA-Fab-cG250.

This fast macroscopic blood clearance observed in the biodistribution studies is clearly shown on the microscopic tumor localization of the Fab at 6 (Figure S10) and 24 h p.i. (Figure 5) where there is a diffuse uptake in the central poorly perfused region of the tumor, which has high CAIX expression. The corresponding digital analysis (Figures 6A and 6B) show much more rapid blood clearance and specific uptake in CAIX rich regions. In particular the 24 hour data (Figure 6B) gives a slope of around -1 for the perfusion correlation and +1 for the CAIX correlation. These images, and the digital analysis, clearly show that the Fab fragment is the most rapid and reliable indicator of CAIX expression in hypoxic tumor areas. However this comes at the cost of around a tenfold reduction in the absolute tumor uptake and a tenfold increase in renal retention of radioactivity.

## Discussion

Imaging of CAIX expression using ^124^I-cG250 has previously been demonstrated to identify accurately clear-cell renal carcinoma (ccRCC), and also provides prognostic information which allows stratification of patients and indicates appropriate treatment options [20]. This PET-based method has great potential for benefit in the diagnosis and management of renal cancer. The high tumor uptake of ^124^I-cG250 in ccRCC relies upon the constitutive high expression of CAIX in this tumor type, which results in high uptake of the radiotracer (7.4±1.8%ID/g at 7 days) and sufficient tumor/blood differential (ca 31) to provide optimal PET images [21].

The expression of CAIX has been shown to have prognostic significance in growing number of tumor types other than ccRCC ([3], [5], [6], [7], [8], [9], [10], [16], [17], [22], [23], [24], [25]). The potential therefore exists to adapt ^124^I-cG250 imaging protocols to allow the monitoring of CAIX expression in a wider range of tumor types. The use of non-invasive PET imaging for evaluation of CAIX expression has several distinct advantages over the histochemical methods currently employed. PET imaging allows absolute quantitation of tracer uptake, which would be preferable to the subjective assessment of CAIX expression made on histological sections. PET imaging also allows for assessments made on the entire tumor volume, as opposed to either biopsy specimens or tissue sections which are inherently subject to sampling errors. Unlike histological evaluation, PET imaging also allows for the possibility of serial imaging, which affords the possibility of monitoring alterations in CAIX expression as an indicator of treatment response.

Solid tumors are know to have a leaky vasculature, with increased permeability to macromolecules[26]. Early work has shown that once the antibody leaves the vascular space, the rate of diffusion within the tumor tissue is highly dependent of the size of the molecule [27], [28]. In addition, diffusion of IgG from well perfused tumor regions may also be limited by a binding site barrier [29]. These observations form the basis for the modeling of the macroscopic uptake IgG, F(ab')_2_ and Fab in tumors [30], [31]. The use of an intact immunoglobulin as the CAIX-targeting moiety for imaging purposes in this setting was originally thought to be limited because of the relatively poor diffusion from the vasculature into and through the tumor [32]. In non ccRCC tumors CAIX is generally associated with areas of hypoxia and necrosis [1], which are by definition relatively long distances from perfused vasculature. Accurate assessment of CAIX expression using non-invasive imaging would require that the issue of penetration of the targeting agent to the site of antigen expression is minimized.

For this study, we generated Fab and F(ab)'_2_ fragments by enzymatic digestion of intact cG250, conjugated each with DOTA, and labeled with ^111^In for evaluation purposes. The radiolabeled IgG, F(ab')_2_ and Fab fragments all demonstrated preserved immunoreactivity. To evaluate the biodistribution and relative tumor penetration of each immunoconjugate, we used subcutaneous xenograft tumors derived from the HT29 human colorectal cell line. This model has a highly inducible CAIX expression under hypoxic conditions [33], and has previously been used as a model of heterogeneous CAIX expression[15]. Apart from visually comparing the endogenous CAIX targeting and tumor perfusion, we refined the pixel by pixel correlation method of digital analysis previously reported by Li et al [15] to give objective analysis of the tumor targeting of the three antibody forms.

Although this study showed that the smaller radiolabeled antibody fragments displayed rapid CAIX targeting when compared to IHC they also showed a much lower absolute uptake compared to the IgG and a very high renal uptake. In terms of reliably reporting CAIX expression, the later images obtained with the intact IgG were comparable to early images with Fab and F(ab')_2_ forms of cG250, and there was a much higher tumor/non tumor contrast which would result in much shorter imaging times. Interestingly the control studies with 3S193 (anti-Lewis-Y) and J591 (anti-PSMA) antibodies at 7 days post injection showed that, apart from some activity in well perfused tumor regions, the highest uptake was observed in areas furthest away from regions of elevated CAIX expression. This likely reflects a combination of non-specific tracer uptake and physical barriers of diffusion into the poorly-perfused, CAIX-expressing tumor regions, rather than an antigen-related distribution. Together, this indicates that the smaller fragments are unlikely to have any additional benefits for the imaging of CAIX with ^124^I-cG250.

This work also demonstrates the application of pixel by pixel correlation for the objective analysis of microscopic tumor antigen targeting. Since all these four images were extracted from the same tumor section, there was a simple and accurate coregistration of all four data sets. The heterogeneous expression of the endogenous CAIX antigen within the single tumor section also serves as a positive and negative control for the in vivo targeted radiolabeled antibodies. The use of the perfusion marker also allows for the measurement of the rates of migration of the radiolabeled antibody into non pefused and CAIX rich regions of the tumor.

### Conclusion

This study demonstrates that intact cG250 IgG can properly report CAIX expression in hypoxic tumors at late imaging time points. The Fab and F(ab')_2_ fragments are capable of targeting CAIX in hypoxic regions at shorter time periods post-injection, but this comes at the cost of reduced absolute uptake and severely reduced tumor to muscle ratios. Since ^124^I-cG250 is in the late stages clinical development, it should serve as a powerful tool to allow for quantitative non-invasive evaluation of CAIX expression in a range of solid tumor types with hypoxia-regulated CAIX expression.

## Materials and Methods

### Materials

The monoclonal antibody cG250 was supplied by the Ludwig Institute for Cancer Research Center (New York, NY). Reagents for generating antibody fragments were from Pierce (Rockfield, IL). All radionuclides were from MDS Nordion (Ottawa, ON Canada). Pimonidazone and FITC-Mab1 were from Hypoxyprobe (Burlington, MA). Alexa-Fluor 569 goat anti human antibody was from Nitrogen (Carlsbad, CA). Hoechst 33342 and all other chemicals were from Sigma-Aldrich (St. Louis, MO). The renal cell carcinoma cell line SKRC38, which has a high endogenous expression of CAIX, was used for in vitro studies and was supplied by Ludwig Institute for Cancer Research (New York, NY). The colorectal adenocarcinoma cell line HT29, which expresses CAIX in vivo, was used for animal studies and was supplied by American Type Culture Collection (Manassas, VA). The CAIX expression of both cells lines were validated during the course of this study.

### Cell culture

SKRC38 cells were grown in RPMI media supplemented with, 1% glutamine, 50 units/ml penicillin, 50 units/ml streptomycin, and 10% heat-inactivated fetal bovine serum and colon HT29 cells were grown in RMPI media supplemented with, 50 units/ml penicillin, 50 units/ml streptomycin, and 10% heat-inactivated fetal bovine serum at a temperature of 37°C in an environment containing 5% CO_2_.

### Generation of Fragments

The antibody cG250 was digested in 20 mM sodium acetate buffer (pH 4.5) with pepsin immobilized on agarose beads to yield F(ab')_2_ fragments [34]. The antibody cG250 was also digested in 20 mM sodium phosphate buffer/10 mM EDTA (pH 7.0) with papain immobilized on agarose beads to yield Fab fragments [34]. The fragment reaction mixtures were partially purified and the buffer exchanged to 10 mM Tris (pH 7.5) using ultrafiltration with a 30 kDa cut off membrane followed by purification with a protein A column to remove undigested IgG and Fc fragments. Final purification of the fragments was performed with a Superdex 200 size exclusion column and an eluant of 50 mM sodium phosphate (pH 7.2). The generated Fab and F(ab')_2_ fragments were labeled with ^131^I using iodogen [35] up to specific activities of 40–120 MBq/mg. They were then purified with size exclusion chromatography with Sephadex G50 and an eluant of PBS with 1% BSA. Radiochemical purity was assessed with ITLC using silica gel impregnated glass fiber strips and an eluant of 5% TFA. The immunoreactivity was determined by the method of Lindmo [36] using SKRC28 cells and saturation binding studies were performed with SKRC38 human renal cancer cells and cell membranes derived from HT29 human colon cancer xenografts [37].

### Antibody Conjugation and Radiolabeling

The intact and fragmented IgG were modified with 1,4,7,10-tetraazacyclododecane-N,N’,N’’,N’’’-tetraacetic acid (DOTA), by direct coupling of one of the four carboxylic acid groups of DOTA to the primary amines in the antibody protein structure. This was achieved by first making an active ester of DOTA using standard peptide coupling reagents of EDC and NHS [37]. The extent of the antibody modification was determined by mixing the conjugate with a known amount indium chloride spiked with ^111^In. Following a chase with DTPA, the amounts of ^111^In-DTPA and ^111^In-mAb were determined by ITLC and the data used to determine the number of binding sites [34]. This data was correlated with the immunoreactivity of the conjugate to select the best agent for *in vivo* imaging.

The antibodies were then labeled with ^111^In using a transfer ligand of acetate [34]. DTPA was used to chelate non-specifically bound radiometals and the radioligands were purified with size exclusion chromatography with Sephadex G50 and an eluant of PBS with 1% BSA. Radiochemical purity was assessed with ITLC using silica gel impregnated glass fiber strips and an eluant of 50 mM DTPA (pH 5.0). The immunoreactivities of the DOTA/antibody conjugates were determined by the method of Lindmo [36]. Briefly, a fixed amount of the radiolabeled antibody was incubated with an increasing number of SKRC38 cells expressing CAIX. The amount of binding was determined and a linear extrapolation of the Total/bound fraction against the inverse number of cells used was performed. The intercept gave the amount of antibody binding at an infinite excess of antigen (i.e. the immunoreactivity).

The radiolabeled ligands were diluted with 5 mM DTPA (pH 7.0) to a concentration of to 3.7 MBq/mL. These test solutions were be stored at 37°C and periodically 10 µL samples removed and analyzed by ITLC using ITLC-SG strips (100×10 mm Pall, Ann Arbor, MI) and a running solvent of 5 mM DTPA (pH 5.0). Under these conditions the antibody remains at the origin and ^111^In-DTPA moves with the solvent front.

### *In Vivo* Evaluation of CAIX Targeting

All animals were handled in strict accordance with good animal practice as defined by the National Institutes of Health (NIH) Guide for the Care and Use of Laboratory Animals, and the Public Health Service (PHS) Policy on Humane Care and Use of Laboratory Animals. Animal protocols were approved by the Institutional Animal Care and Use Committee at Memorial Sloan-Kettering Cancer Center (MSKCC). Prior to use, HT29 cells were trypsinized, counted and suspended phosphate buffered saline (binding studies) with 50% Matrigel for tumor implantation. Six- to eight-week old *nu/nu* athymic male mice were maintained in ventilated cages and fed/watered *ad libertum* and the experiments were be carried out under an IACUC approved protocol as well as following institutional guidelines for the proper and humane use of animals in research. 1×10^7^ tumor cells were injected sc into the flanks of the animals and after 14–21 days tumors (50–200 mg) had developed.

Tumor bearing mice were injected, via the tail vein, with 1.6 MBq of an ^111^In labeled ligand in 200 µL of PBS (pH 7.4, 0.2% BSA). Selected animals were also injected with Pimomidazole hydrochloride (60 mg/kg, hypoxyprobe-1, HPI, Burlington, MA) one hour pre sacrifice to assess tumor hypoxia and Hoechst 33342 (40 mg/kg) at 5 minutes pre sacrifice to assess tumor perfusion. Groups of animals (n = 4–5) were sacrificed, by CO_2_ euthanasia, after 6 hours, 1, 2,4, or 7 days post injection of the radiotracer. Tumor and tissue samples were collected, weighed and counted, with appropriate standards, in an automatic NaI(Tl) counter. These measured relative activity data (cpm) were background corrected and expressed as a percentage of the injected dose per gram (%ID/g). These data were also fitted with a least squares regression analysis to determine the biological clearance of the different forms of antibody. The tumors were immediately frozen at −80°C and sections processed for immunohistochemistry and or digital autoradiography.

### Immunofluorescence staining and imaging

For CAIX staining, tumor sections were fixed for 12 min in 4% paraformaldehyde solution and blocked using Superblock blocking buffer in PBS, (Pierce, Rockford, IL) at room temperature for 1 hr. Sections are then incubated with primary antibody (cG250, a murine Fv grafted human IgG1K - kindly supplied by Ludwig Institute of Cancer Research) applied in blocking solution at a concentration of 25 µg/ml. Slides were incubated for 1 hr at room temperature, washed three times with PBS and incubated with secondary fluorescent Alex-568 goat anti-human antibody in blocking solution at a concentration of 40 µg/ml. Negative controls consisted of tumor sections exposed to fluorescent anti-human antibody alone. Pimonidazole distribution was determined by incubation of blocked sections with anti-pimonidazole-FITC conjugate (HPI, 1∶10 dilution in Superblock) at for 1 hour at room temperature.

Tumor sections were then imaged with a Zeiss Axioplan2 fluorescence microscope connected to a CCD camera in RGB mode. Fluorescence images were obtained using appropriate filter cubes for each fluorochrome. The microscope has a computer controlled motorized stage that enables images of whole sections to be generated as a mosaic at any given magnification with identical exposure time per frame.

### Digital Autoradiography

Tumor sections were exposed to a phosphor-imaging plate (Fujifilm BAS-MS2325, Fuji Photo Film, Japan) for an appropriate length of time at -20°C. Upon the completion of an exposure, the imaging plates were removed from the cassette and placed in a BAS-1800II Bio-Imaging Analyzer (Fujifilm Medical Systems, USA) to readout the image. The image reader creates 16-bit grayscale digital images with pixel size of 50 µm. These images are then converted to. tiff image format files for subsequent analysis.

### Image registration and analysis

All four image sets were rescaled to a resolution of 18 µm/pixel and coregistered using visual tumor landmarks (e.g. tumor rim). The four separate layers were exported as tiff image files. These files were loaded into Origin (Microcal, Northhampton, MA) and the image data converted to a single column of values. The four columns of data were loaded into Excel spreadsheet (Microsoft) and the data sorted in ascending order according to the coregistered ^111^In activity. This sorted data was collated into 20 equally sized bins according increasing ^111^In values. The binned data was then plotted as a function of the mean ^111^In data.

Statistical comparisons for the mean uptake in an organ between the various experimental groups were performed by one-way ANOVA with Bonferroni's multiple comparison post-hoc test. All statistical comparisons were two sided and the level of statistical significance set at p<0.05.

## Supporting Information

Figure S1 Binding properties of radiolabeled cG250. Figure 1A: Saturation binding of ^111^In-DOTA-cG250 to SKRC38 human renal cancer cells, Figure 1B: Saturation binding of ^111^In-DOTA-F(ab')_2_-cG250 to SKRC38 human renal cancer cells, Figure 1C: Saturation binding of ^111^In-DOTA-Fab-cG250 to SKRC38 human renal cancer cells, Figure 1D: Lindmo immunoreactivity testing of ^111^In-DOTA-cG250 with SKRC38 renal cancer cell. The y intercept of 1.11 indicates an immunoreactivity of 90% at an infinite antigen excess.
(7.24 MB TIF)

Figure S2 Tumor/non-tumor ratios for ^111^In labeled intact and fragmented cG250 in athymic mice with hypoxic HT29 colorectal tumors. A: ^111^In-DOTA-cG250. B: ^111^In-DOTA-F(ab')_2_-cG250. C: ^111^In-DOTA-Fab-cG250. Legend Bl: blood, He: heart, Lu: lungs, Sp: spleen, Ki: kidneys, St: stomach, S.I.: small intestine, L.I.: large intestine and Mu: muscle.
(6.25 MB TIF)

Figure S3 Top panel: HT29 tumor sections showing blood perfusion (*in vivo* targeted Hoechst, blue), microscopic biodistribution of ^111^In-DOTA-cG250 (*in vivo* targeted, white) and endogenous CAIX (*ex vivo* cG250 immunofluorescence, red) at 4 days pi. Bottom panel: Enlarged 3×3 mm areas of tumor sections.
(6.00 MB TIF)

Figure S4 Top panel: HT29 tumor sections showing blood perfusion (*in vivo* targeted Hoechst, blue), microscopic biodistribution of ^111^In-DOTA-cG250 (*in vivo* targeted, white) and endogenous CAIX (*ex vivo* cG250 immunofluorescence, red) at 7 days pi. Bottom panel: Enlarged 3×3 mm areas of tumor sections.
(5.63 MB TIF)

Figure S5 Top panel: HT29 tumor sections showing blood perfusion (*in vivo* targeted Hoechst, blue), microscopic biodistribution of ^111^In-DOTA-3S193 (*in vivo* targeted, white) and endogenous CAIX (*ex vivo* cG250 immunofluorescence, red) at 7 days pi. Bottom panel: Enlarged 3×3 mm areas of tumor sections.
(6.28 MB TIF)

Figure S6 Top panel: HT29 tumor sections showing blood perfusion (*in vivo* targeted Hoechst, blue), microscopic biodistribution of ^111^In-DOTA-J591 (*in vivo* targeted, white) and endogenous CAIX (*ex vivo* cG250 immunofluorescence, red) at 7 days pi. Bottom panel: Enlarged 3×3 mm areas of tumor sections.
(5.78 MB TIF)

Figure S7 Binned pixel by pixel analysis of the correlation of ^111^In-DOTA-IgG *in vivo* uptake with endogenous CAIX expression (*ex vivo* cG250 immunofluorescence) and tumor perfusion (*in vivo* targeted with Hoechst) in HT29 colorectal tumors at 7 days p.i. for 3S193 (A) and J591 (B) control antibodies.
(4.83 MB TIF)

Figure S8 Top panel: HT29 tumor sections showing blood perfusion (*in vivo* targeted Hoechst, blue), microscopic biodistribution of ^111^In-DOTA-F(ab')_2_-cG250 (*in vivo* targeted, white) and endogenous CAIX (*ex vivo* cG250 immunofluorescence, red) at 6 hours pi. Bottom panel: Enlarged 3×3 mm areas of tumor sections.
(5.59 MB TIF)

Figure S9 Top panel: HT29 tumor sections showing blood perfusion (*in vivo* targeted Hoechst, blue), microscopic biodistribution of ^111^In-DOTA-F(ab')_2_-cG250 (*in vivo* targeted, white) and endogenous CAIX (*ex vivo* cG250 immunofluorescence, red) at 24 hours pi. Bottom panel: Enlarged 3×3 mm areas of tumor sections.
(5.67 MB TIF)

Figure S10 Top panel: HT29 tumor sections showing blood perfusion (*in vivo* targeted Hoechst, blue), microscopic biodistribution of ^111^In-DOTA-Fab-cG250 (*in vivo* targeted, white) and endogenous CAIX (*ex vivo* cG250 immunofluorescence, red) at 6 hours pi. Bottom panel: Enlarged 3×3 mm areas of tumor sections.
(5.76 MB TIF)
