# Supplementary figures and images for: Molecular Targeting of Carbonic Anhydrase IX in Mice with Hypoxic HT29 Colorectal Tumor Xenografts
Source: PLoS One. 2010 May 27;5(5):e10857. doi: 10.1371/journal.pone.0010857 (PMC2877709; doi:10.1371/journal.pone.0010857)

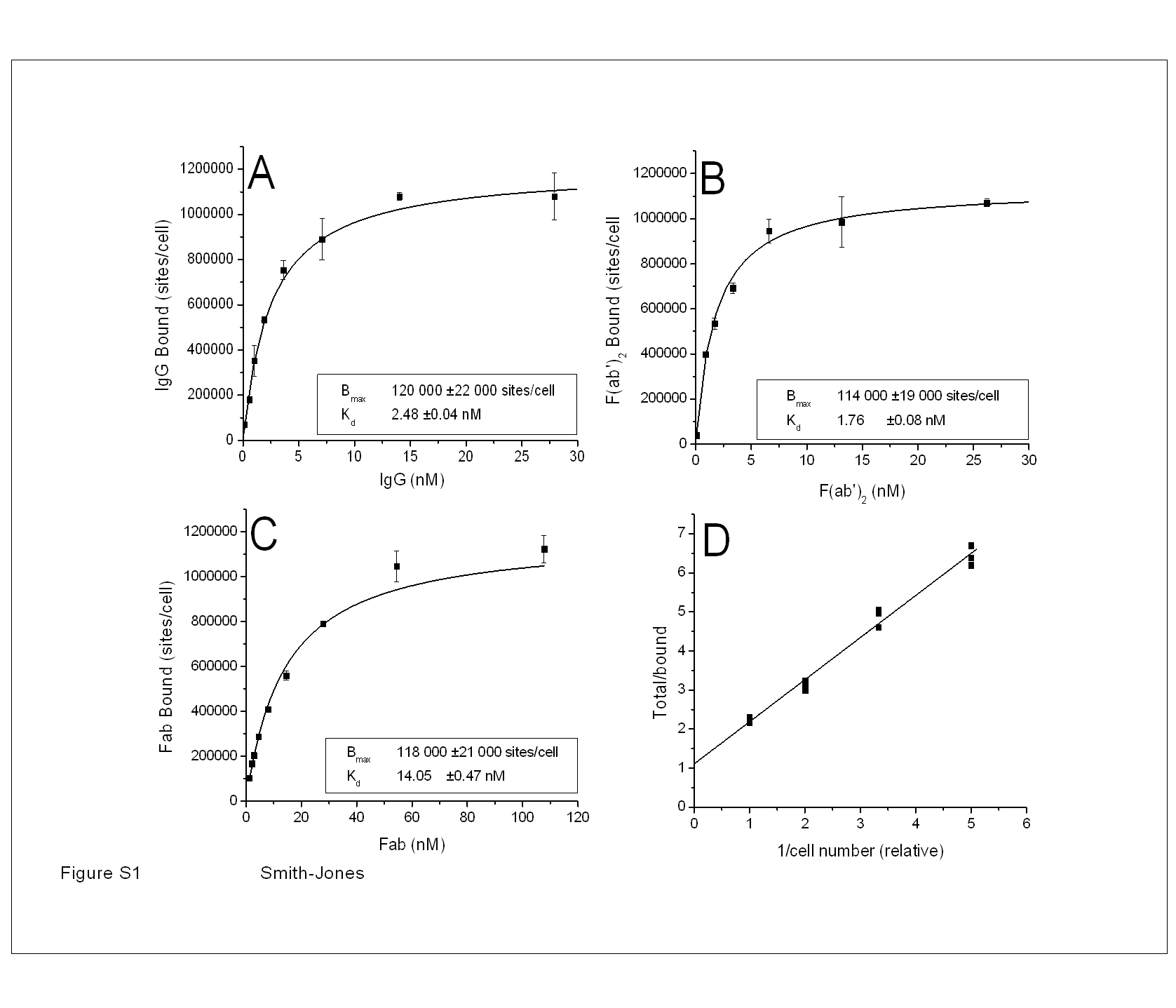

Supplement: Figure S1 — Binding properties of radiolabeled cG250. Figure 1A: Saturation binding of 111In-DOTA-cG250 to SKRC38 human renal cancer cells, Figure 1B: Saturation binding of 111In-DOTA-F(ab')2-cG250 to SKRC38 human renal cancer cells, Figure 1C: Saturation binding of 111In-DOTA-Fab-cG250 to SKRC38 human renal cancer cells, Figure 1D: Lindmo immunoreactivity testing of 111In-DOTA-cG250 with SKRC38 renal cancer cell. The y intercept of 1.11 indicates an immunoreactivity of 90% at an infinite antigen excess. (7.24 MB TIF) [file pone.0010857.s001.tif]

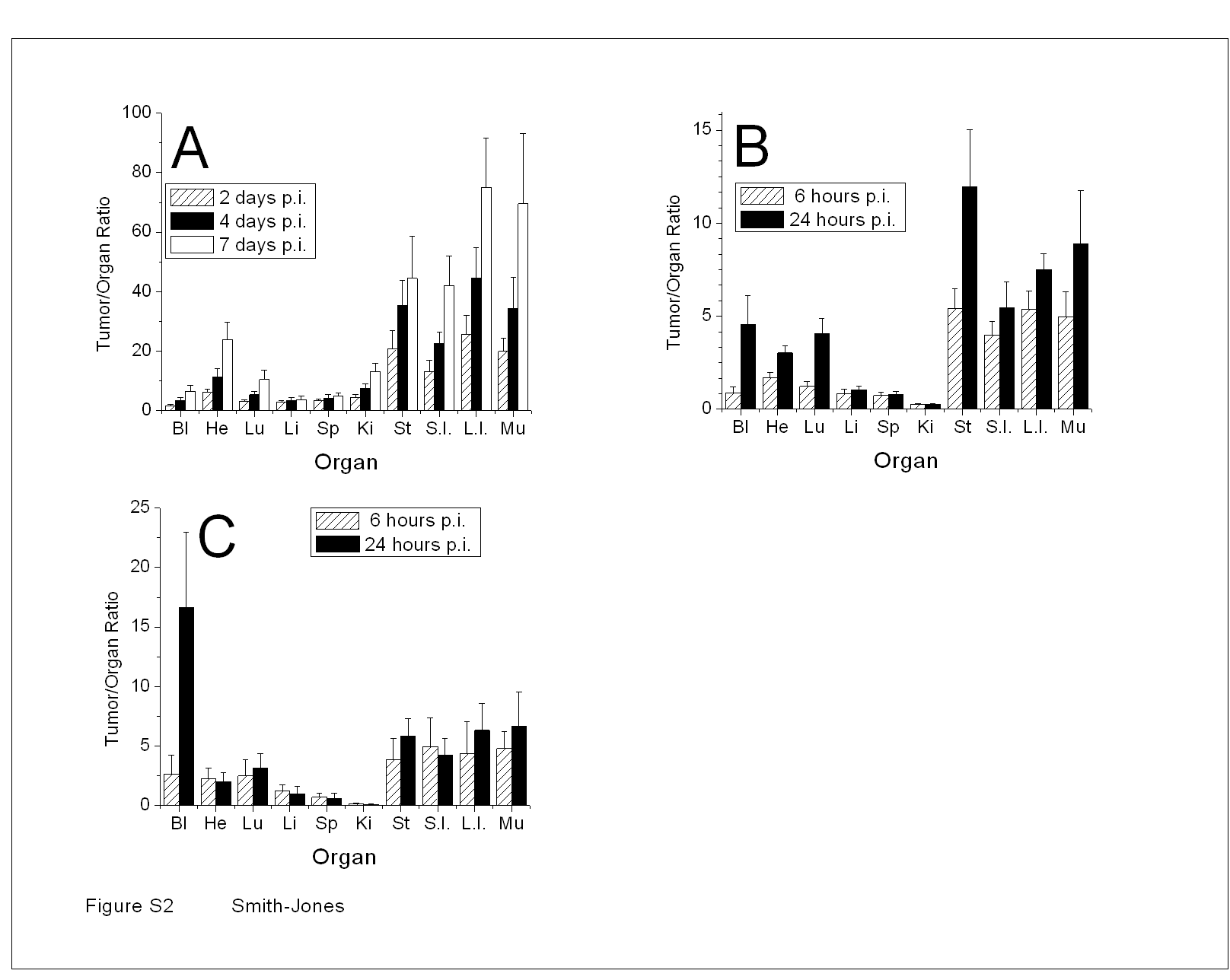

Supplement: Figure S2 — Tumor/non-tumor ratios for 111In labeled intact and fragmented cG250 in athymic mice with hypoxic HT29 colorectal tumors. A: 111In-DOTA-cG250. B: 111In-DOTA-F(ab')2-cG250. C: 111In-DOTA-Fab-cG250. Legend Bl: blood, He: heart, Lu: lungs, Sp: spleen, Ki: kidneys, St: stomach, S.I.: small intestine, L.I.: large intestine and Mu: muscle. (6.25 MB TIF) [file pone.0010857.s002.tif]

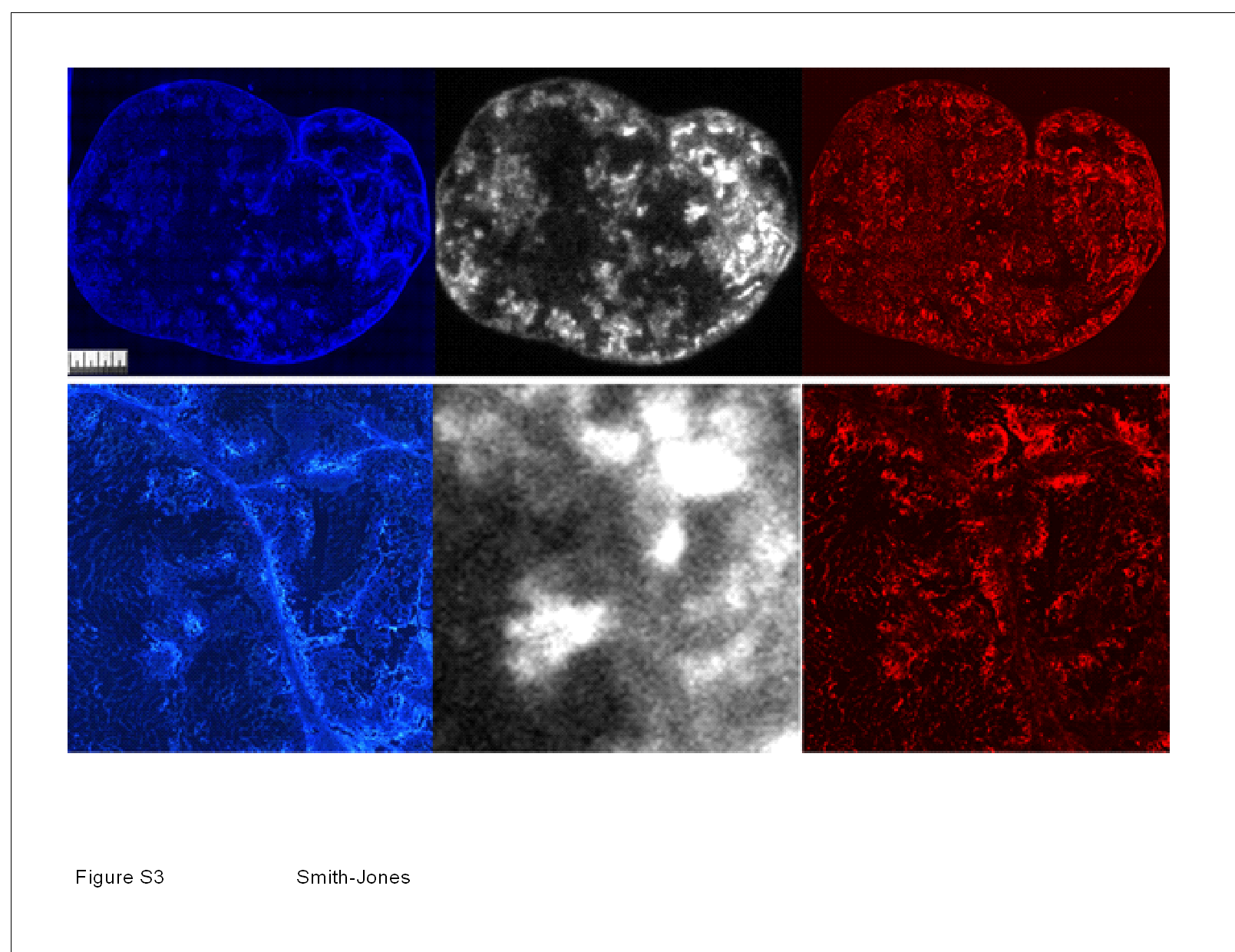

Supplement: Figure S3 — Top panel: HT29 tumor sections showing blood perfusion (in vivo targeted Hoechst, blue), microscopic biodistribution of 111In-DOTA-cG250 (in vivo targeted, white) and endogenous CAIX (ex vivo cG250 immunofluorescence, red) at 4 days pi. Bottom panel: Enlarged 3×3 mm areas of tumor sections. (6.00 MB TIF) [file pone.0010857.s003.tif]

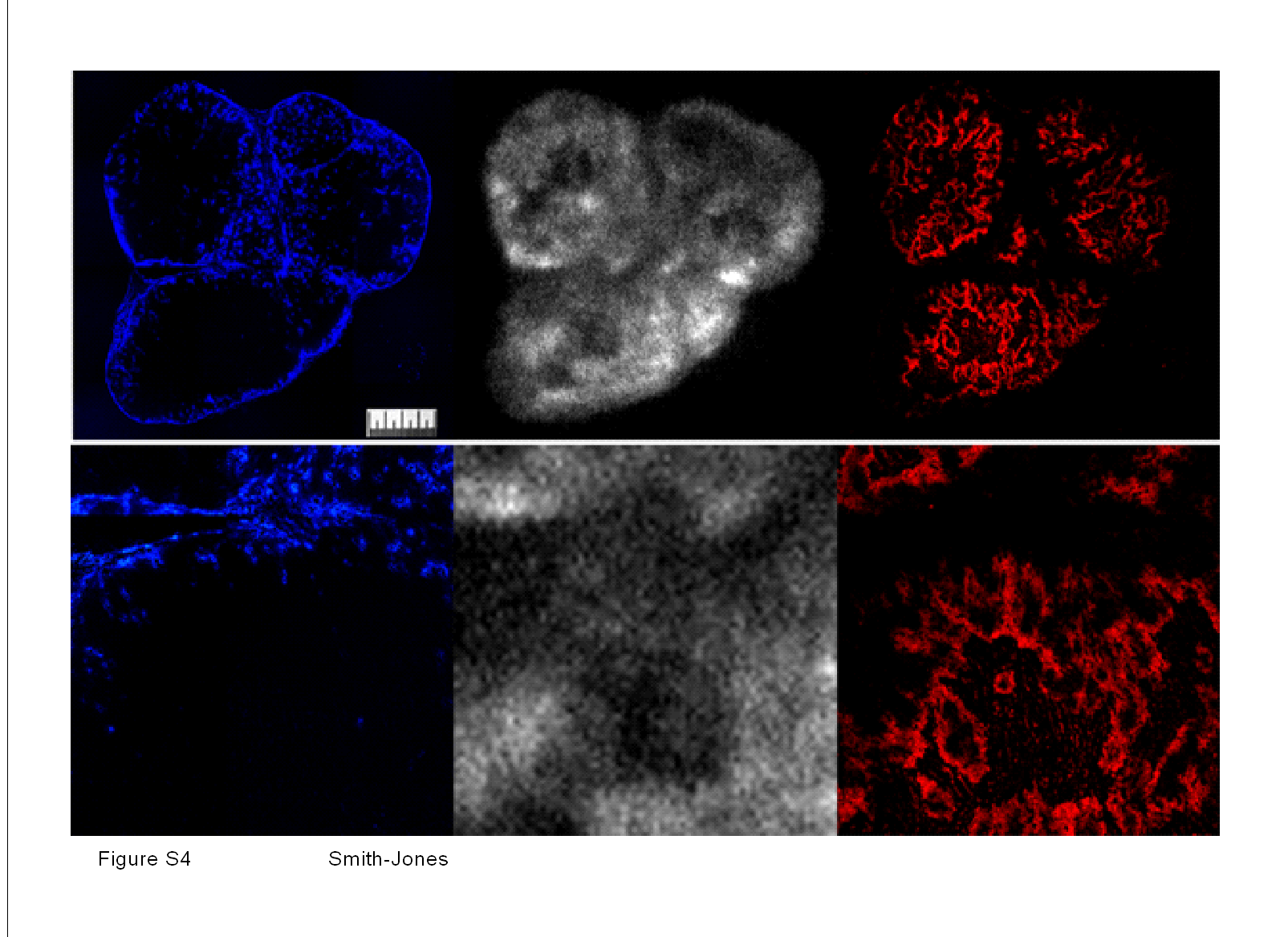

Supplement: Figure S4 — Top panel: HT29 tumor sections showing blood perfusion (in vivo targeted Hoechst, blue), microscopic biodistribution of 111In-DOTA-cG250 (in vivo targeted, white) and endogenous CAIX (ex vivo cG250 immunofluorescence, red) at 7 days pi. Bottom panel: Enlarged 3×3 mm areas of tumor sections. (5.63 MB TIF) [file pone.0010857.s004.tif]

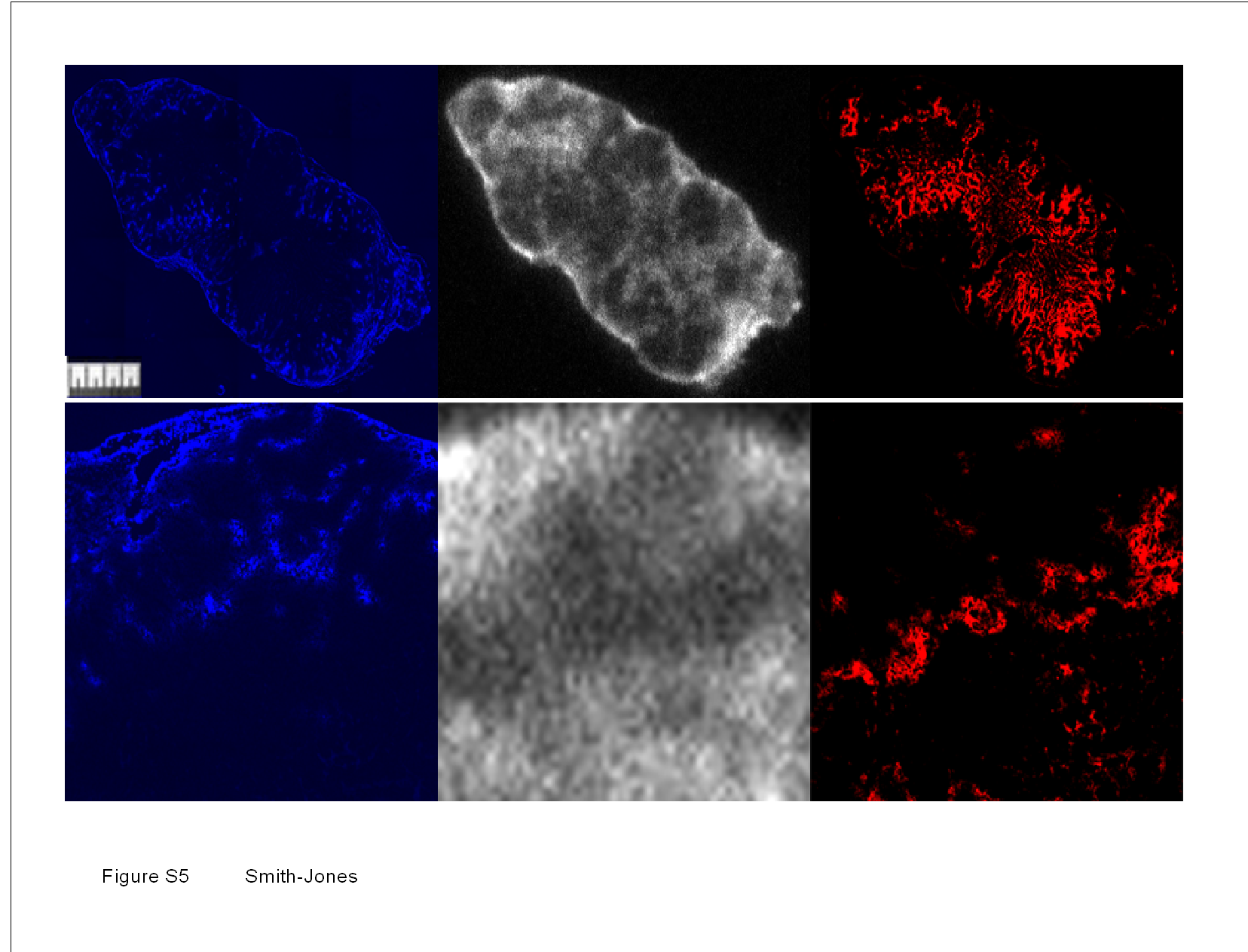

Supplement: Figure S5 — Top panel: HT29 tumor sections showing blood perfusion (in vivo targeted Hoechst, blue), microscopic biodistribution of 111In-DOTA-3S193 (in vivo targeted, white) and endogenous CAIX (ex vivo cG250 immunofluorescence, red) at 7 days pi. Bottom panel: Enlarged 3×3 mm areas of tumor sections. (6.28 MB TIF) [file pone.0010857.s005.tif]

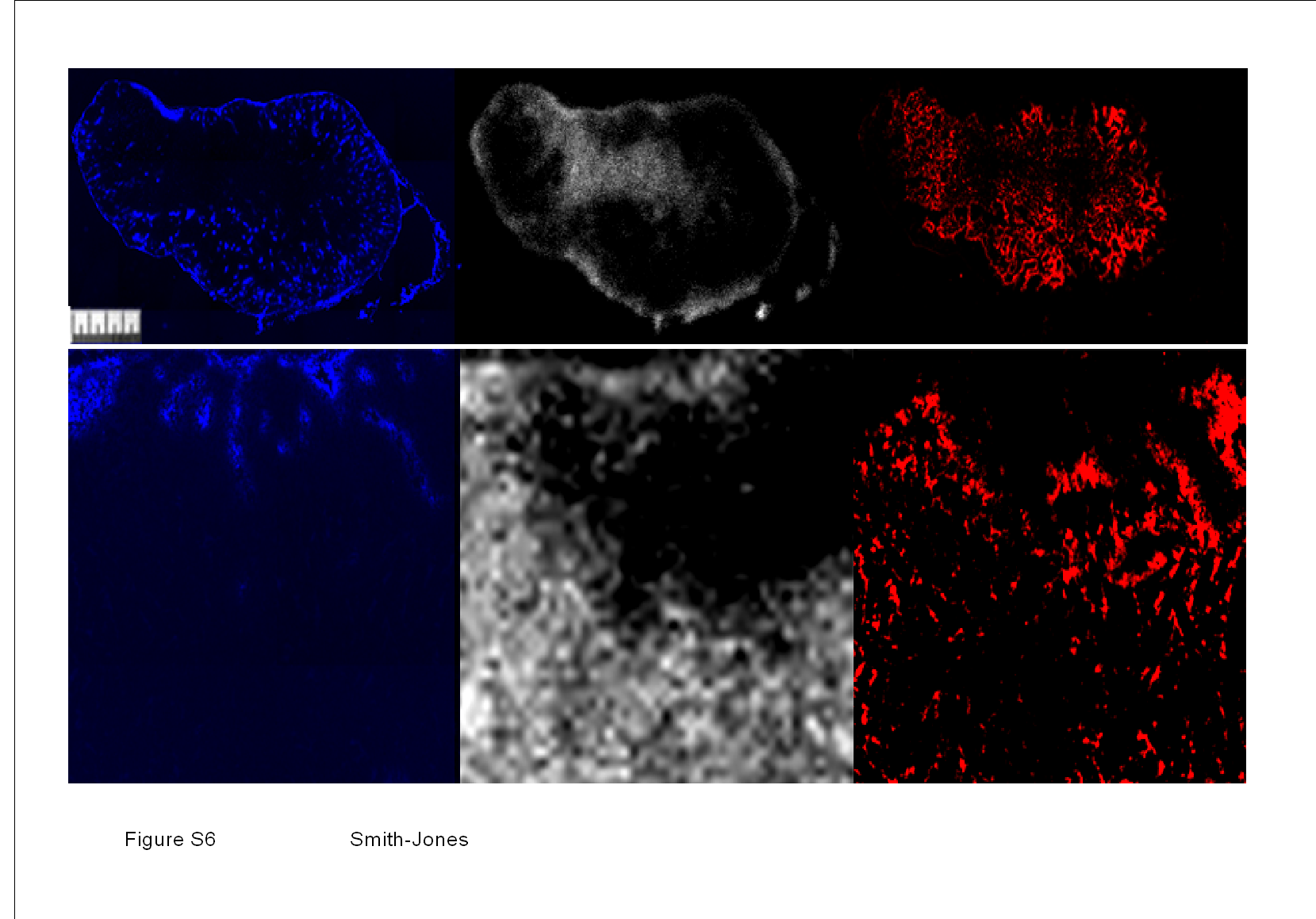

Supplement: Figure S6 — Top panel: HT29 tumor sections showing blood perfusion (in vivo targeted Hoechst, blue), microscopic biodistribution of 111In-DOTA-J591 (in vivo targeted, white) and endogenous CAIX (ex vivo cG250 immunofluorescence, red) at 7 days pi. Bottom panel: Enlarged 3×3 mm areas of tumor sections. (5.78 MB TIF) [file pone.0010857.s006.tif]

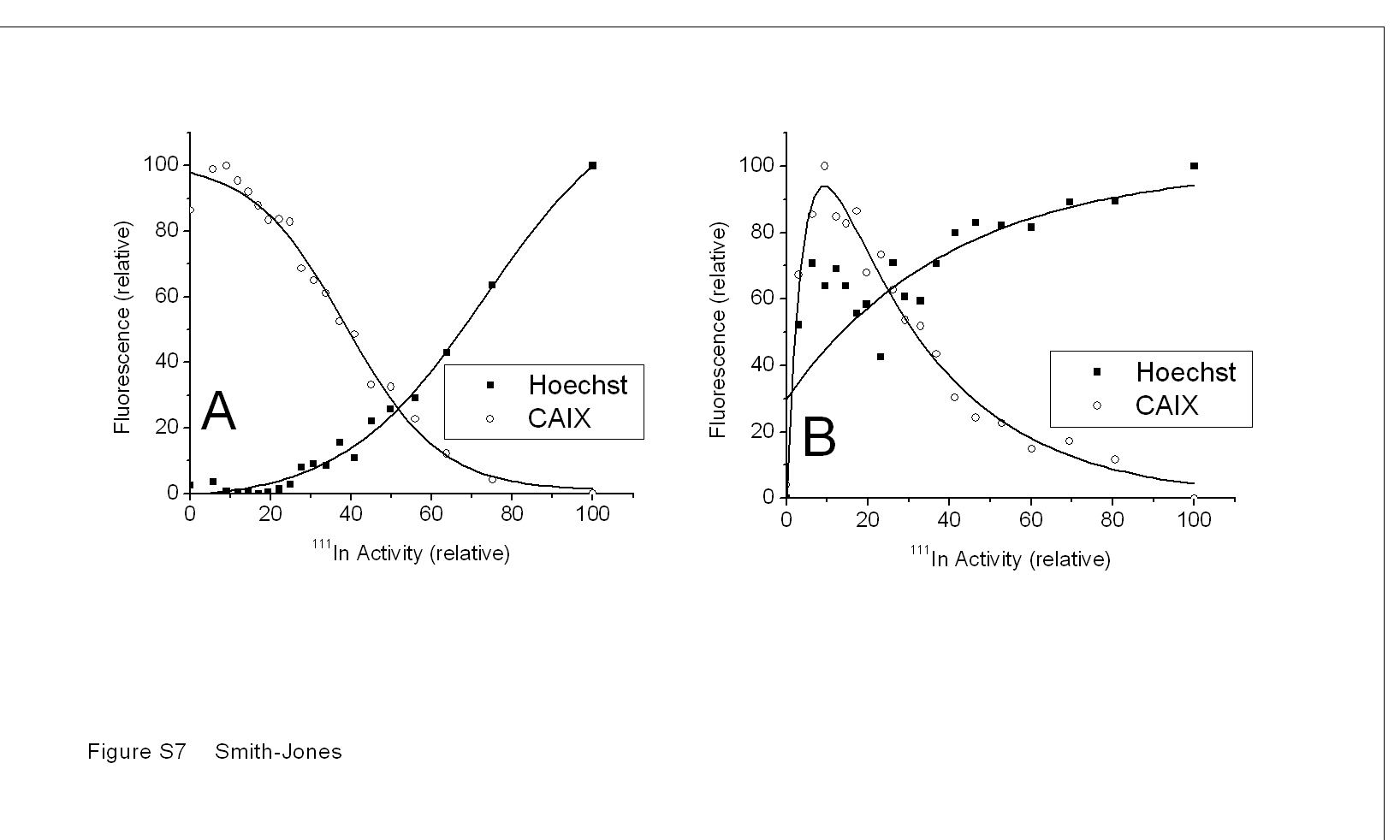

Supplement: Figure S7 — Binned pixel by pixel analysis of the correlation of 111In-DOTA-IgG in vivo uptake with endogenous CAIX expression (ex vivo cG250 immunofluorescence) and tumor perfusion (in vivo targeted with Hoechst) in HT29 colorectal tumors at 7 days p.i. for 3S193 (A) and J591 (B) control antibodies. (4.83 MB TIF) [file pone.0010857.s007.tif]

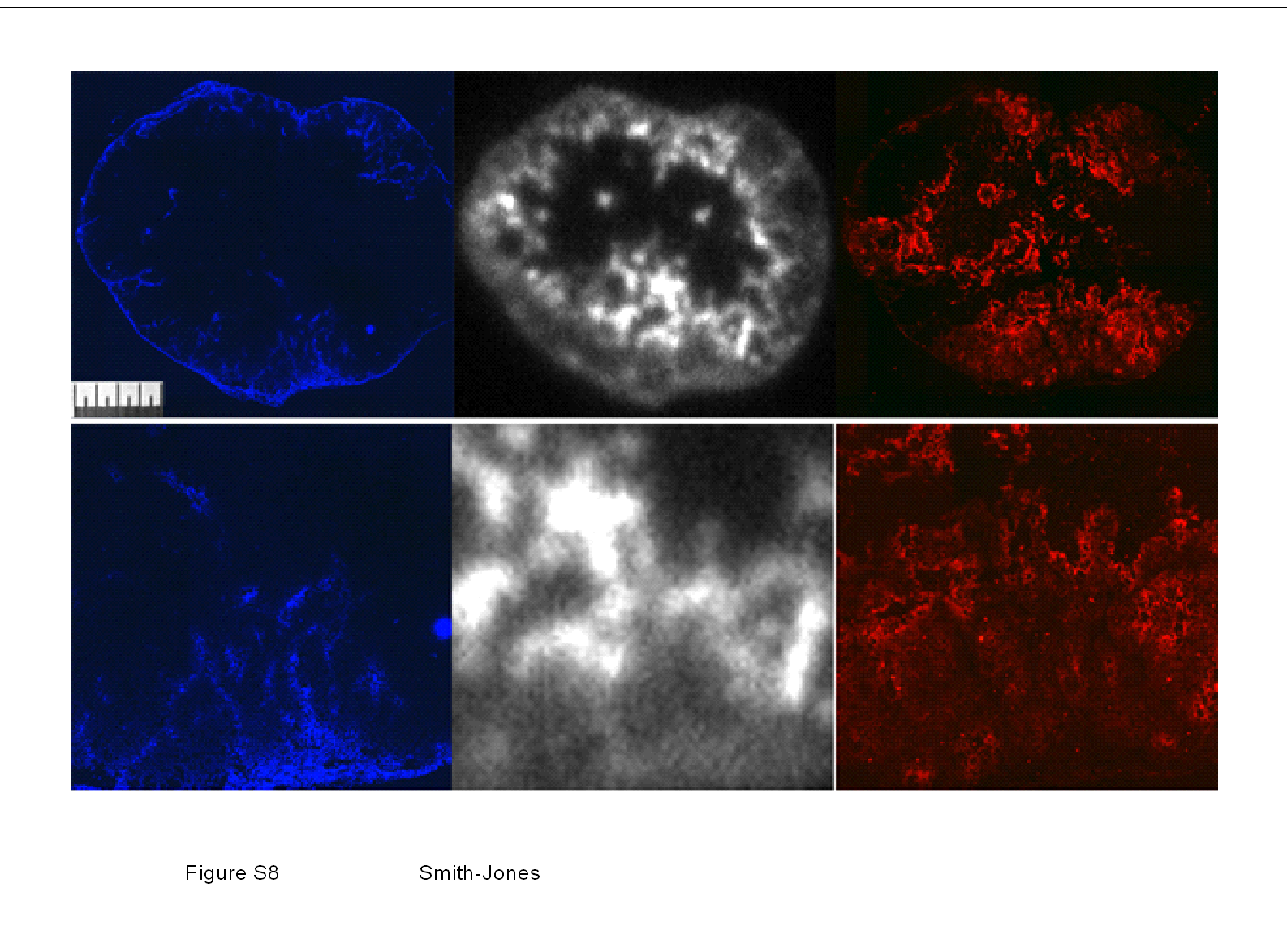

Supplement: Figure S8 — Top panel: HT29 tumor sections showing blood perfusion (in vivo targeted Hoechst, blue), microscopic biodistribution of 111In-DOTA-F(ab')2-cG250 (in vivo targeted, white) and endogenous CAIX (ex vivo cG250 immunofluorescence, red) at 6 hours pi. Bottom panel: Enlarged 3×3 mm areas of tumor sections. (5.59 MB TIF) [file pone.0010857.s008.tif]

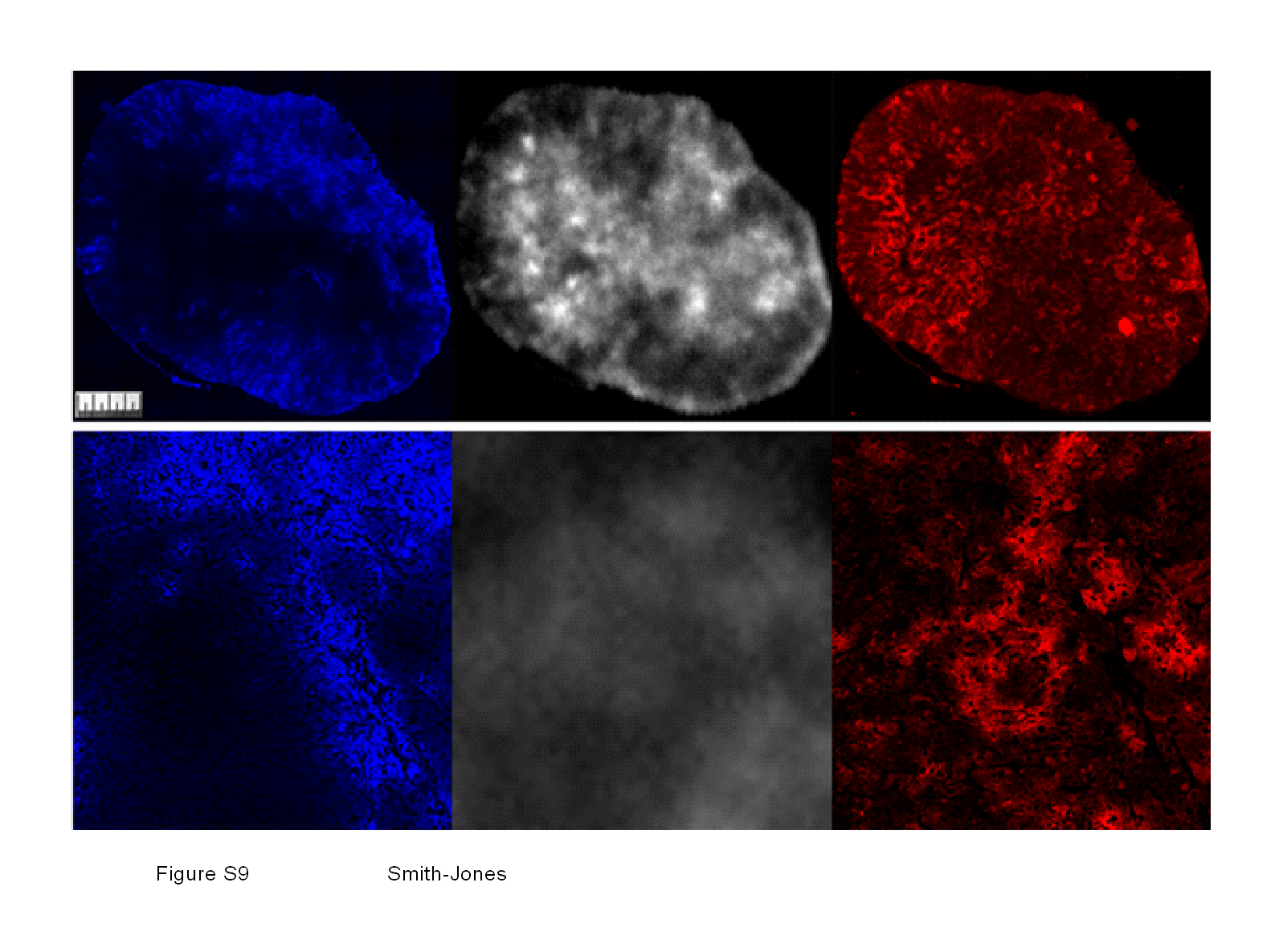

Supplement: Figure S9 — Top panel: HT29 tumor sections showing blood perfusion (in vivo targeted Hoechst, blue), microscopic biodistribution of 111In-DOTA-F(ab')2-cG250 (in vivo targeted, white) and endogenous CAIX (ex vivo cG250 immunofluorescence, red) at 24 hours pi. Bottom panel: Enlarged 3×3 mm areas of tumor sections. (5.67 MB TIF) [file pone.0010857.s009.tif]

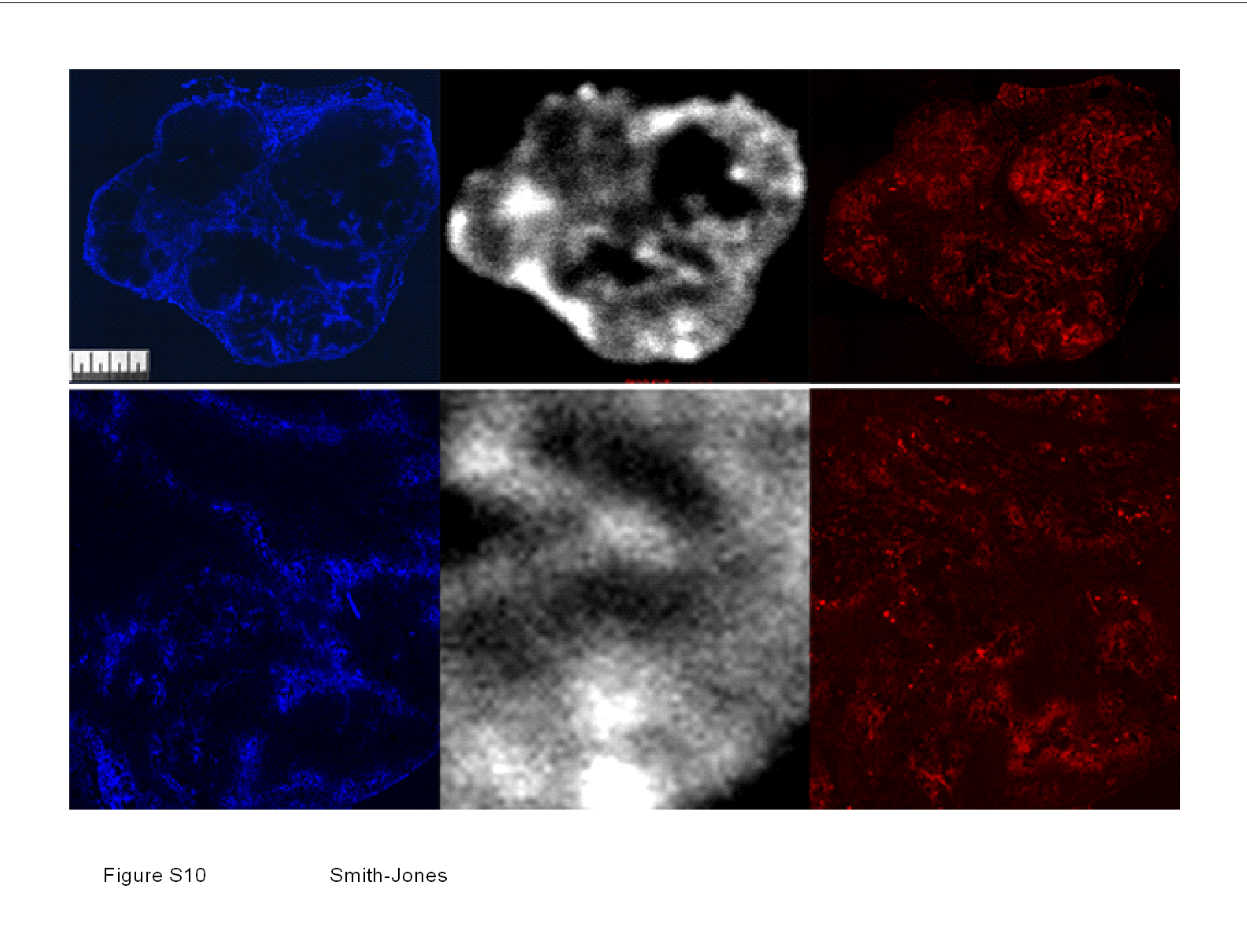

Supplement: Figure S10 — Top panel: HT29 tumor sections showing blood perfusion (in vivo targeted Hoechst, blue), microscopic biodistribution of 111In-DOTA-Fab-cG250 (in vivo targeted, white) and endogenous CAIX (ex vivo cG250 immunofluorescence, red) at 6 hours pi. Bottom panel: Enlarged 3×3 mm areas of tumor sections. (5.76 MB TIF) [file pone.0010857.s010.tif]
